# Enlarged perivascular spaces are associated with decreased brain tau deposition

**DOI:** 10.1111/cns.14040

**Published:** 2022-12-05

**Authors:** Koung Mi Kang, Min Soo Byun, Dahyun Yi, Kyung Hoon Lee, Min Jung Kim, Hyejin Ahn, Gijung Jung, Jun‐Young Lee, Yu Kyeong Kim, Yun‐Sang Lee, Chul‐Ho Sohn, Dong Young Lee, for the KBASE Research Group

**Affiliations:** ^1^ Department of Radiology Seoul National University Hospital Seoul Korea; ^2^ Department of Radiology Seoul National University College of Medicine Seoul Korea; ^3^ Department of Neuropsychiatry Seoul National University College of Medicine Seoul Korea; ^4^ Department of Neuropsychiatry Seoul National University Hospital Seoul Korea; ^5^ Biomedical Research Institute, Seoul National University Hospital Seoul Korea; ^6^ Department of Neuropsychiatry SMG‐SNU Boramae Medical Center Seoul Korea; ^7^ Department of Nuclear Medicine SMG‐SNU Boramae Medical Center Seoul Korea; ^8^ Department of Nuclear Medicine Seoul National University College of Medicine Seoul Korea; ^9^ Institute of Human Behavioral Medicine Medical Research Center Seoul National University Seoul Korea

**Keywords:** Alzheimer's disease, amyloid PET, enlarged perivascular spaces, tau PET

## Abstract

**Aims:**

The aim of this study was to investigate the associations of enlarged perivascular spaces (EPVS) in the basal ganglia (BG) and centrum semiovale (CSO) with beta‐amyloid (Aβ) and tau deposition in older adults with a diverse cognitive spectrum.

**Methods:**

A total of 163 (68 cognitively normal and 95 cognitively impaired) older participants underwent [^11^C] Pittsburgh compound B and [^18^F] AV‐1451 PET, and MRI. EPVS in the BG and CSO and other small vessel disease markers, such as white matter hyperintensities, lacunes, and deep and lobar microbleeds, were assessed.

**Results:**

Increased EPVS in the BG showed a significant association with lower cerebral tau deposition, even after controlling for other small vessel disease markers. Further exploratory analyses showed that this association was significant in cognitively impaired, Aβ‐positive, or APOE4‐positive individuals, but not significant in the cognitively normal, Aβ‐negative, or APOE4‐negative participants. In contrast to EPVS in the BG, EPVS in the CSO did not have any relationship with cerebral tau deposition. In addition, none of the two types of EPVS were associated with cerebral Aβ deposition.

**Conclusion:**

Brain tau deposition appears to be reduced with increased EPVS in the BG, especially in individuals with cognitive impairment, pathological amyloid burden, or genetic Alzheimer's disease risk.

## Graphical abstract

Enlarged perivascular spaces in the basal ganglia were associated with decreased tau burden, especially in individuals with cognitive impairment, pathological amyloid burden, or genetic Alzheimer's disease risk. In contrast to the enlarged perivascular spaces in the basal ganglia, enlarged perivascular spaces in the centrum semiovale did not associate with cerebral tau burden.

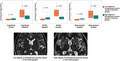

## 1 INTRODUCTION

Enlarged perivascular spaces (EPVS) are fluid‐filled spaces that surround the penetrating small vessels in the brain and accord with extensions of the subarachnoid space.^1^ Enlarged perivascular spaces are typically observed in the basal ganglia (BG) and can also be seen in the centrum semiovale (CSO).^1^ There are increasing reports that PVS are elements of the “glymphatic system,” which plays an important role in the clearance of interstitial fluid (ISF) and waste from the brain through interstitial fluid (ISF)‐mediated bulk flow.^2^, ^3^, ^4^, ^5^ Increased visibility of EPVS on brain MRI has been associated with various neurological conditions such as degenerative diseases and stroke.^6^

Several previous studies have reported associations between EPVS and clinically defined Alzheimer's disease dementia or mild cognitive impairment (MCI). Chen et al.^7^ reported significant associations between overall EPVS and the diagnosis of Alzheimer's disease dementia or MCI. Hansen et al.^8^ reported that EPVS in the BG (BG‐EPVS) were more prevalent in Alzheimer's disease dementia than in normal controls, while other studies demonstrated that the EPVS in the CSO (CSO‐EPVS), rather than the BG‐EPVS, were more closely associated with Alzheimer's disease dementia compared with normal control^9^ or subcortical vascular cognitive impairment.^10^

Nevertheless, the relationship between EPVS and the neuropathological hallmarks of Alzheimer's disease, that is, cerebral beta‐amyloid (Aβ) and tau deposition, has not been clearly understood. Some neuroimaging studies reported a positive association between CSO‐EPVS and Aβ deposition in individuals with various cognitive spectrums,^11^, ^12^ while others found no such association.^10^, ^13^, ^14^ With regard to the relationship between EPVS and tau pathology, only limited and controversial information is yet available. One recent study on cognitively normal (CN) individuals showed that CSO‐EPVS, but not BG‐EPVS, was related to tau PET positivity,^14^ while another study on cognitively diverse individuals did not find an association between EPVS in any location and CSF tau levels in individuals without dementia.^13^

In this context, we aimed to investigate the association of regional EPVS, that is, CSO‐EPVS and BG‐EPVS, with in vivo cerebral Aβ and tau deposition measured by PET in older adults with a diverse cognitive spectrum including CN and cognitively impaired (CI) individuals (i.e., MCI and Alzheimer's disease dementia).

## 2 METHODS

### 2.1 Participants

A total of 163 older adults (68 CN and 95 CI [45 MCI and 50 Alzheimer's disease dementia]), who participated in the Korean Brain Aging Study for Early Diagnosis and Prediction of Alzheimer's Disease (KBASE) study,^15^ were included. The CN participants had no diagnosis of MCI or dementia, and a Clinical Dementia Rating score (CDR) of 0. Participants with MCI had a global CDR of 0.5, and fulfilled the core clinical criteria for diagnosis of MCI according to the recommendations of the National Institute on Aging‐Alzheimer's Association guidelines (NIA‐AA).^16^ Participants with Alzheimer's disease dementia had a global CDR score of 0.5 or 1 and met the criteria for dementia in accordance with the Diagnostic and Statistical Manual of Mental Disorders 4th Edition (DSM‐IV‐TR), and the criteria for probable Alzheimer's disease dementia in accordance with the NIA‐AA.^17^ Individuals with a major psychiatric illness, significant neurological or medical condition, or comorbidities that could affect mental function were excluded from the study. Details of participant recruitment and the inclusion or exclusion criteria were described in our previous report.^15^ The study protocol was approved by the Institutional Review Boards of Seoul National University Hospital and SNU‐SMG Boramae Center in Seoul, South Korea; the participants or their legal representatives provided written informed consent.

### 2.2 Clinical assessment

All participants underwent comprehensive clinical and neuropsychological assessments by trained psychiatrists and neuropsychologists based on the KBASE assessment protocol, which incorporates the Consortium to Establish a Registry for Alzheimer's Disease (CERAD‐K).^15^ Blood sampling was completed to determine apolipoprotein E ε4 allele (APOE4) carrier status. A vascular risk score was calculated based on some vascular risk factors, including hypertension, diabetes mellitus, hyperlipidemia, coronary artery disease, transient ischemic attack, and stroke, reported as a percentage.^18^

### 2.3 Neuroimaging data

All participants underwent simultaneous three‐dimensional [^11^C] Pittsburgh compound B (PiB) PET and MRI, including three‐dimensional (3D) T1‐weighted images, 3D fluid‐attenuated inversion‐recovery (FLAIR) images, T2‐weighted images, and susceptibility‐weighted images using a 3.0 T Biograph mMR (PET‐MR) Scanner (Siemens, Washington DC, USA). Three‐dimensional T1‐weighted images and FLAIR images were acquired in the sagittal plane. Acquisition parameters for 3D T1‐weighted images were as follows: repetition time (TR), 1670 ms; echo time (TE), 1.89 ms; field of view (FOV), 250 mm; matrix, 256 × 256; slice thickness, 1.0 mm. The parameters for acquiring 3D FLAIR images were as follows: TR, 5000 ms; TE, 173 ms; echo spacing, 3.46 ms; FOV, 250 mm; matrix size, 256 × 256; slice thickness, 1.0 mm. Acquisition parameters for T2‐weighted images were as follows: TR, 5000 ms; TE, 91 ms; FOV, 199 × 200 mm; matrix size, 640 × 348; and slice thickness, 3.0 mm. Acquisition parameters for susceptibility‐weighted images were as follows: TR, 1670 ms; TE, 1.9 ms; FOV, 250 mm; matrix size, 448 × 255; and slice thickness, 3.0 mm. All participants also underwent [^18^F] AV‐1451 PET scans using a Biograph True Point 40 PET/CT Scanner (Siemens, Washington DC, USA).

### 2.4 Assessment of enlarged perivascular spaces and other small vessel diseases

Enlarged perivascular spaces, cerebral microbleeds, and lacunes were visually detected by a consensus of two board‐certified neuroradiologists (KMK and KHL) blinded to the clinical information. Enlarged perivascular spaces were defined as small, sharply delineated structures of <3 mm in size, with signal intensity similar to CSF, that follows the course of perforating vessels, and are visually assessed from T2‐weighted images according to the rating scale by Potter et al. (ed.ac.uk/files/imports/fileManager/epvs‐rating‐scale‐user‐guide.pdf). Both BG‐EPVS and CSO‐EPVS were rated using a validated five‐point visual rating scale (0 = none, 1 = 1–10, 2 = 11–20, 3 = 21–40, and 4 = > 40 EPVS).^19^, ^20^ After assessing all relevant slices for the anatomical area, the highest number of EPVS was recorded (above the anterior commissure for BG‐EPVS).^13^ Due to the fact that both BG‐EPVS and CSO‐EPVS were not normally distributed, the degree of each EPVS was dichotomized into low (degree 0–1) and high (degree 2–4), as also used previously.^13^, ^14^, ^21^, ^22^ Cerebral microbleeds were defined and counted on the susceptibility‐weighted images and divided into lobar or deep cerebral microbleeds based on location.^23^ Focal round hypointensities between 1 and 10 mm in diameter and distinguished from vessels were considered cerebral microbleeds. Lacunes ranging in size from 3 to 15 mm were identified with a combination of T1‐weighted, T2‐weighted, and FLAIR images if the lesion had a signal of CSF with a perilesional halo on FLAIR images.^1^ Interobserver agreements were excellent for the assessment of the five‐point visual rating scale of EPVS (kappa values, 0.81 for BG‐EPVS and 0.94 for CSO‐EPVS), the number of deep cerebral microbleeds (intraclass correlation coefficient = 0.890 [95% confidence interval: 0.853, 0.918]), lobar cerebral microbleeds (0.997 [0.996, 0.998]), and lacunes (0.939 [0.917, 0.954]). The volume of white matter hyperintensities on FLAIR images was calculated using a validated automatic procedure^24^ with minor modifications. We used an optimal threshold of 70 for our data instead of the recommended 65 from the original study,^24^ as it is more suitable for our scans obtained using mMR Biograph PET‐MR. White matter hyperintensities candidate images were used to extract white matter hyperintensities volumes based on lobar regions of interest (ROIs) in the native space for each participant.^25^

### 2.5 Assessment of beta‐amyloid deposition

After intravenous administration of 555 MBq of [^11^C] PiB (range, 450–610 MBq), a 30‐min emission scan was obtained 40 min after injection using the 3.0 T PET‐MR scanner mentioned above. The PiB‐PET data collected in list mode were processed for routine corrections, including uniformity, UTE‐based attenuation, and decay corrections, and were reconstructed into a 344 × 344 image matrix using iterative methods of five iterations with 21 subsets. The image preprocessing steps were performed using Statistical Parametric Mapping 8 (SPM8; http://www.fil.ion.ucl.ac.uk/spm) implemented in MATLAB 2014a (MathWorks, Natick, MA, USA). For each participant, static PiB‐PET images were co‐registered with individual T1‐weighted images. Transformation parameters were calculated for spatial normalization of individual T1 images to a standard Montreal Neurological Institute (MNI) template. The inverse transformation of parameters was used to transform coordinates from the automatic anatomic labeling (AAL) 116 atlas^26^ into an individual space for each participant (resampling voxel size = 1 × 0.98 × 0.98 mm) using the Individual Brain Atlases using SPM (IBMSPM) software in MATLAB. A gray matter mask, which is a binary probabilistic gray matter map generated by a preprocessing step using SPM8, was applied to each individual to extract the gray matter and exclude the non‐gray matter portions of the atlas (i.e., white matter and CSF space). The mean regional PiB uptake values from the cerebral regions were extracted using the individual AAL116 atlas from the T1‐co‐registered PiB‐PET images. Cerebellar gray matter was used as the reference region because of its relatively low Aβ deposition,^27^ with a probabilistic cerebellar atlas (Institute of Cognitive Neuroscience, UCL; Cognitive Neuroscience Laboratory, Royal Holloway, University of London, UK). To characterize the PiB retention levels, the AAL algorithm and a region combining method^28^ were used to determine ROIs in the frontal, lateral parietal, posterior cingulate‐precuneus, and lateral temporal regions. The global cerebral Aβ retention value (standardized uptake value ratio [SUVR]) was calculated by dividing the voxel‐weighted mean value of the four ROIs by the mean cerebellar uptake value.^28^, ^29^, ^30^ Each participant was classified as Aβ‐positive if the global cerebral Aβ retention value was >1.4, or Aβ‐negative if the global Aβ retention value was ≤1.4 in at least one of the abovementioned four ROIs.^28^, ^29^, ^30^

### 2.6 Assessment of tau deposition

For the measurement of cerebral tau deposition, a 20‐min emission scan was obtained 80 min after injection of 370 MBq of [^18^F] AV‐1451 on a Biograph True point 40 PET/CT Scanner (Siemens) as dynamic scans using LIST mode. The participants underwent low‐dose CT scans in the same position immediately prior to the PET scans for attenuation correction. The iterative (OSEM3D+PSF) True X algorithm was used for PET data reconstruction with 24 subsets, six iterations with a 3‐mm Gaussian filter. To measure tau protein, [^18^F] AV‐1451 PET SUVR images were created based on the mean uptake over 80–100 min post‐injection, normalized by the mean inferior cerebellar gray matter uptake, and then co‐registered and resliced into structural MRIs. The Geometric Transfer Matrix approach for partial volume correction was applied based on FreeSurfer‐derived ROIs from the T1‐weighted images taken at the follow‐up visit, including corrections for extracerebral tissue as described previously.^31^, ^32^ For the ROI analyses, the data were extracted from native space according to the method published by Baker et al.^31^ We quantified [^18^F] AV‐1451 PET uptake by grouping together ROIs that corresponded to the pathological stages of tau protein tangle deposition in Alzheimer's disease, as described by Braak and Braak.^33^ The regions grouped in each Braak stage region of interest have been published previously.^31^, ^34^ For the current analysis, a cerebral global tau ROI mean was used; the global ROI consists of the regions corresponding to anatomical definitions of Alzheimer's disease Braak stages I/II, III/IV, and V/VI.

### 2.7 Statistical analysis

Baseline characteristics are expressed as mean ± standard deviation or median (range) for continuous variables and as numbers (percentages) for categorical variables. Normality of data was determined using the Shapiro–Wilk test. Comparisons were made between the CN and CI participants using the chi‐squared test for categorical variables, independent *t*‐test for normally distributed continuous variables, and Mann–Whitney U‐test for continuous variables that were not normally distributed. The associations between each of the dichotomous measures of BG‐EPVS and CSO‐EPVS (as independent variables) and each of global cerebral Aβ and tau deposition (as a dependent variable) were analyzed using two general linear models. Model 1 included age, sex, cognitive status (CN or CI) as covariates, and APOE4 carrier status. Model 2 included the number of deep cerebral microbleeds, lobar cerebral microbleeds, lacunes, and white matter hyperintensities volume as additional covariates, as well as the covariates included in Model 1. The Bonferroni correction method was applied to perform multiple comparisons using *p* < 0.025 (=0.05/2. i.e., No. of analyses within each dependent variable). In addition, for exploratory purposes, when any relationship between EPVS and cerebral Aβ or tau deposition was significant, the moderation effects of age, sex, cognitive status, and APOE4 carrier status were investigated using the general linear model analysis, including each of the variables × EPVS interaction term as an additional independent variable. If there was a significant association between EPVS and tau deposition, the moderation effect of Aβ positivity was also explored by including Aβ positivity × EPVS interaction term as an additional independent variable in the model. When any interaction term in the models was significant, subsequent subgroup analyses were performed using an additional general linear model for each subgroup divided by moderation variables. For the exploratory analyses, *p* < 0.05 was applied. All statistical analyses were performed using IBM SPSS Statistics 23 (SPSS Inc., Chicago, IL, USA). Statistical significance was set at *p* < 0.05, unless otherwise specified.

## 3 RESULTS

### 3.1 Characteristics of the participants

The demographic and clinical characteristics of the participants are presented in Table 1. High‐degree BG‐EPVS was more prevalent in the CI group than in the CN group, while CSO‐EPVS did not differ between the two groups (Table 1 and Figure S1).

**TABLE 1:** Demographic and clinical characteristics

Variables	All (*n* = 163)	CI subjects (*n* = 95)	CN subjects (*n* = 68)	*p*‐value
Mean age (y)	73.2 ± 7.2	74.3 ± 7.2	71.6 ± 7.1	0.017*
Females, *n* (%)	108 (66.3)	67 (70.5)	41 (60.3)	0.173
APOE4 carriers	59 (36.2)	47 (49.5)	12 (17.6)	<0.001*
Vascular risk factor score	16.7 (0–66.7)	16.7 (0–66.7)	16.7 (0–66.7)	0.459
Global CDR (0.5/1)	57 (35)/34 (21)	57 (60)/34 (36)	0 (0)/0 (0)	<0.001*
CDR– Sum of box	1 (0–11)	3.5 (0.5–11)	0 (0–0.5)	<0.001*
Cerebral Aβ deposition, SUVR	1.38 (0.81–2.80)	1.76 (0.81–2.80)	1.14 (0.81–1.63)	<0.001*
Aβ positivity	86 (52.8)	64 (67.4)	22 (32.4)	<0.001*
Cerebral tau deposition, SUVR	1.29 (0.65–4.32)	1.50 (0.65–4.32)	1.18 (0.81–1.63)	<0.001*
Dichotomous measures of EPVS				
BG‐EPVS				
Low (0–1)	65 (39.9)	30 (31.6)	35 (51.5)	0.011*
High (2–4)	98 (60.1)	65 (68.4)	33 (48.5)	
CSO‐EPVS				
Low (0–1)	27 (16.6)	14 (14.7)	13 (19.1)	0.458
High (2–4)	136 (83.4)	81 (85.3)	55 (80.9)	
Deep cerebral microbleed count	0 (0–3)	0 (0–3)	0 (0–2)	0.148
Lobar cerebral microbleed count	0 (0–109)	0 (0–109)	0 (0–3)	0.007*
Number of lacunes	0 (0–3)	0 (0–3)	0 (0–3)	0.415
White matter hyperintensities volume (cm^3^)	9.08 (0–88.99)	8.61 (0–88.99)	9.34 (0–57.79)	0.987

*Note*: Data are presented as *n* (%), mean ± standard deviation for normally distributed variables or median (interquartile ranges), min–max for non‐normally distributed variables. Comparisons between CN and CI subjects were performed using the chi‐squared test for categorical variables, independent *t*‐test for normally distributed continuous variables, and Mann–Whitney *U*‐test for non‐normally distributed continuous variables.

Abbreviations: Aβ, beta‐amyloid; APOE4, apolipoprotein E ε4 allele; BG‐EPVS, enlarged perivascular spaces in the basal ganglia; CDR, clinical dementia rating; CI, cognitively impairment; CN, cognitively normal; CSO‐EPVS, enlarged perivascular spaces in the centrum semiovale; SUVR, standardized uptake value ratio.

* *p* < 0.05.

### 3.2 Association of EPVS with cerebral Aβ and tau deposition

**FIGURE 1:**
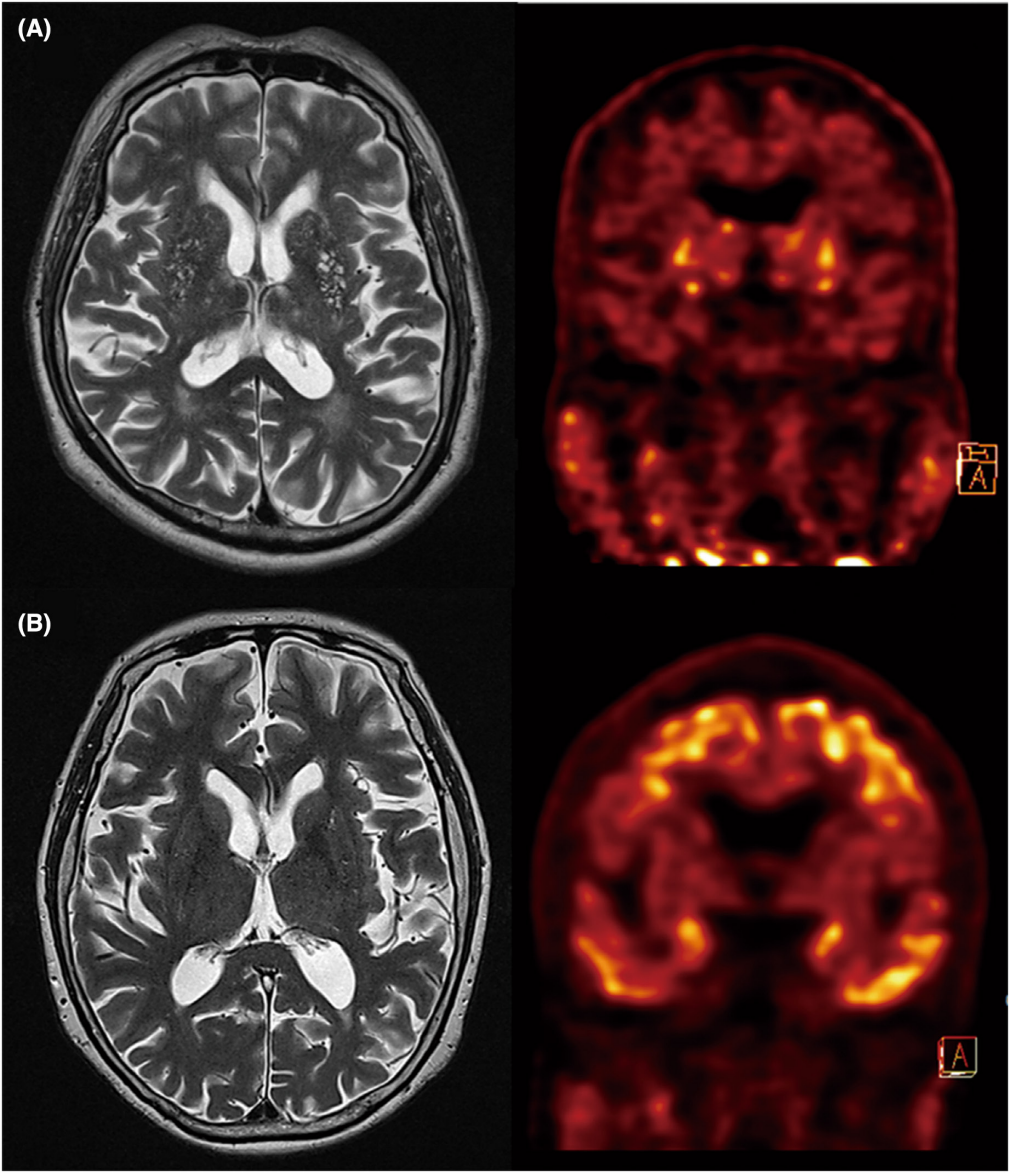
T2‐weighted MR images and tau PET images in two representative cases. (A) a 79‐year‐old man with high‐degree of BG‐EPVS and low brain tau deposition (0.1 SUVR). (B) a 68‐year‐old woman with low degree of BG‐EPVS and high brain tau deposition (4.25 SUVR). BG‐EPVS, enlarged perivascular spaces in the basal ganglia; SUVR, standardized uptake value ratio.

For all participants, there was a significant negative association between BG‐EPVS and cerebral tau deposition (*p* = 0.009 for model 1), even after additionally controlling for the deep cerebral microbleeds, lobar cerebral microbleeds, number of lacunes, and white matter hyperintensities volume (*p* = 0.01 for model 2; Table 2 and Figure 1). By contrast, no significant association was observed between CSO‐EPVS and cerebral tau deposition. Neither BG‐EPVS nor CSO‐EPVS had a significant association with cerebral Aβ deposition, regardless of the model (Table 2).

**TABLE 2:** Associations of EPVS with Cerebral Aβ and tau deposition in overall participants

AD biomarker	Dichotomous EPVS measures	Model 1			Model 2		
		B	SE	*p*‐value	B	SE	*p*‐value
Cerebral Aβ							
	BG‐EPVS	−0.158	0.078	0.045	−0.133	0.078	0.090
	CSO‐EPVS	0.035	0.097	0.715	0.032	0.096	0.741
Cerebral tau							
	BG‐EPVS	−0.258	0.098	0.009*	−0.251	0.099	0.013*
	CSO‐EPVS	0.040	0.122	0.744	0.065	0.123	0.600

*Note*: Model 1 was adjusted for age, sex, cognitive status (CN or CI), and APOE4 carrier status. Model 2 was adjusted for model 1 and small vessel disease markers: deep cerebral microbleed count, lobar cerebral microbleed count, number of lacunes, and white matter hyperintensities volume.

Abbreviations: Aβ, beta‐amyloid; APOE4, apolipoprotein E ε4 allele; BG‐EPVS, enlarged perivascular spaces in the basal ganglia; CI, cognitively impairment; CN, cognitively normal; CSO‐EPVS, enlarged perivascular spaces in the centrum semiovale; SE, standard error.

* Bonferroni corrected *p* < 0.05/2 = 0.025 was used as a statistical threshold.

### 3.3 Moderation of age, gender, APOE4, cognitive status, and Aβ positivity on the relationship between BG‐EPVS and cerebral tau deposition

**FIGURE 2:**
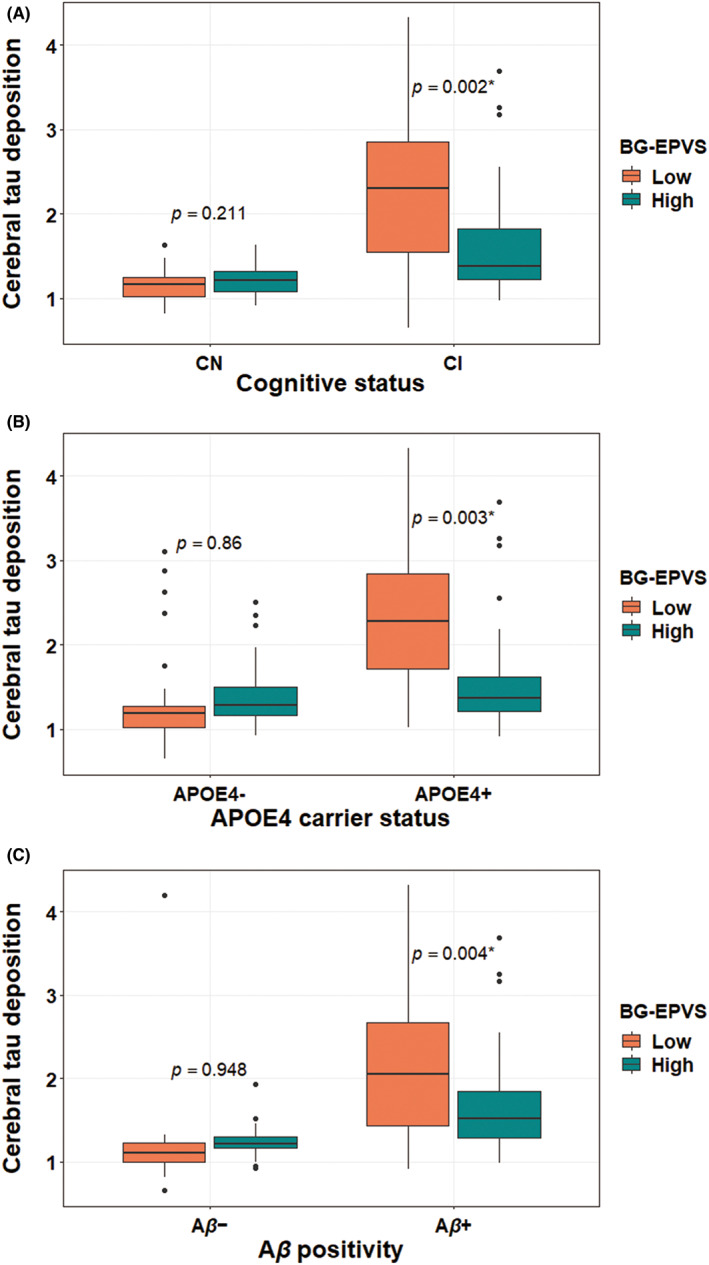
Comparison of cerebral tau deposition between low degree and high degree of BG‐EPVS by (A) cognitive status, (B) APOE4 carrier status, and (C) Aβ positivity status in all older adults. The bar plots represent the average cerebral tau deposition. *p < 0.05. BG‐EPVS, enlarged perivascular spaces in the basal ganglia; APOE4, apolipoprotein E ε4 allele; Aβ, beta‐amyloid.

Considering the relationship between BG‐EPVS and cerebral tau deposition, which showed statistical significance, we further explored the moderating effect of age, sex, cognitive status, APOE4 carrier status, and Aβ positivity using the general linear models, including each of them × BG‐EPVS interaction term. As shown in Table 3, we found significant cognitive status × BG‐EPVS (*p* < 0.001), APOE4 × BG‐EPVS (*p* < 0.001), and Aβ positivity × BG‐EPVS (*p* = 0.006) interaction effects on tau deposition, while no significant interaction effect was found between age or sex and BG‐EPVS on tau deposition. Subsequent subgroup analyses revealed significant negative associations between BG‐EPVS and cerebral tau deposition in CI (*p* = 0.002), APOE4‐positive (*p* = 0.003), and Aβ‐positive individuals (*p* = 0.004), but not in CN (*p* = 0.21), APOE4‐negative (*p* = 0.86), and Aβ‐negative participants (*p* = 0.95) (Table 4, Figure 2, and Figure S2).

**TABLE 3:** Interaction Effect between BG‐EPVS and each of age, sex, cognitive status, APOE4 carrier status, and Aβ positivity on cerebral tau deposition in overall participants

Variables	B	SE	*p*‐value
BG‐EPVS	−0.238	0.119	0.047*
Age^a^	−0.140	0.160	0.384
Age × BG‐EPVS^a^,^b^	−0.077	0.199	0.698
BG‐EPVS	−0.285	0.115	0.015*
Sex	−0.035	0.150	0.816
Sex × BG‐EPVS^c^	0.084	0.191	0.660
BG‐EPVS	0.10	0.132	0.451
Cognitive status	0.980	0.139	<0.001*
Cognitive status × BG‐EPVS^d^	−0.684	0.177	<0.001*
BG‐EPVS	0.010	0.113	0.928
APOE4 carrier status	0.797	0.144	<0.001*
APOE4 carrier status × BG‐EPVS^e^	−0.740	0.178	<0.001*
BG‐EPVS	0.030	0.128	0.817
Aβ positivity	0.738	0.142	<0.001*
Aβ positivity × BG‐EPVS^f^	−0.467	0.166	0.006*

Abbreviations: Aβ, beta‐amyloid; APOE4, apolipoprotein E ε4 allele; BG‐EPVS, enlarged perivascular spaces in the basal ganglia; CI, cognitive impairment; CN, cognitively normal; CSO‐EPVS, enlarged perivascular spaces in the centrum semiovale; EPVS, enlarged perivascular spaces; SE, standard error.

* *p* < 0.05.

^a^ Age indicates a dichotomous measure of age (<75 years or ≥75 years).

^b^ Adjusted for sex, APOE4 carrier status, and cognitive status (CN or CI).

^c^ Adjusted for age, APOE4 carrier status, and cognitive status.

^d^ Adjusted for age, sex, and APOE4 carrier status.

^e^ Adjusted for age, sex, and cognitive status.

^f^ Adjusted for age, sex, and cognitive status and APOE4 carrier status.

**TABLE 4:** Results from the subgroup analyses for relationships of BG‐EPVS with cerebral tau deposition

Cognitive status						
	CN (*n* = 68)			CI (*n* = 95)		
Variable	B	SE	*p*‐value	B	SE	*p‐*value
BG‐EPVS^a^	0.060	0.047	0.211	−0.533	0.166	0.002*

APOE4 carrier status						
	APOE4 non‐carriers (*n* = 104)			APOE4 carriers (*n* = 59)		
Variable	B	SE	*p*‐value	B	SE	*p*‐value
BG‐EPVS^b^	−0.015	0.085	0.860	−0.645	0.204	0.003*

Aβ positivity						
	Aβ‐negative (*n* = 77)			Aβ‐positive (*n* = 86)		
Variable	B	SE	*p*‐value	B	SE	*p*‐value
BG‐EPVS^c^	−0.006	0.099	0.948	−0.416	0.140	0.004*

Abbreviations: Aβ, beta‐amyloid; APOE4, apolipoprotein E ε4 allele; BG‐EPVS, enlarged perivascular spaces in the basal ganglia; CI, cognitive impairment; CN, cognitively normal; CSO‐EPVS, enlarged perivascular spaces in the centrum semiovale.

* *p* < 0.05.

^a^ Adjusted for age, sex, and APOE4 carrier status.

^b^ Adjusted for age, sex, and cognitive status (CN or CI).

^c^ Adjusted for age, sex, cognitive status, and APOE4 carrier status.

## 4 DISCUSSION

In our study, BG‐EPVS had a significant negative association with cerebral tau deposition, even after controlling for other small vessel disease markers. Further exploratory analyses showed that the association between increased BG‐EPVS and lower tau deposition was prominent in CI, APOE4‐positive, or Aβ‐positive individuals, but not in CN, APOE4‐negative, or Aβ‐negative individuals. In contrast to BG‐EPVS, CSO‐EPVS did not have any relationship with cerebral tau deposition. In addition, neither BG‐EPVS nor CSO‐EPVS were associated with cerebral Aβ deposition.

The findings of this study on the relationship between increased BG‐EPVS and lower cerebral tau deposition is a novel one, in contrast to the finding of no association between BG‐EPVS and Aβ level. The contrast between tau and Aβ deposition in relation to BG‐EPVS may be explained by the different clearance systems of the brain for the two proteins. Aβ is cleared from the brain via blood–brain barrier (BBB) transport, degradation, ISF bulk flow to the perivascular space, and CSF absorption.^4^ In particular, BBB transport and the perivascular ISF pathway are responsible for the clearance of Alzheimer's disease‐related Aβ from the brain, covering 80–85% and 15–20%, respectively.^4^, ^35^ By contrast, tau is removed mainly via degradation, the perivascular ISF pathway, and CSF absorption because it cannot be transported across the BBB.^4^ The role of the glymphatic system, which includes drainage of ISF to the perivascular space, is increasingly recognized for the clearance of these proteins.^4^, ^36^ Accumulating evidence suggests that BBB impairment associated with cerebrovascular dysfunction and neuroinflammation occurs before the development of cognitive impairment and before detectable increases in Aβ and tau deposition.^37^, ^38^, ^39^ According to the two‐hit vascular hypothesis of Alzheimer's disease, damage to blood vessels is the initial insult through BBB dysfunction and diminished brain perfusion, which promote Aβ accumulation. BBB breakdown was also reported to be pronounced in APOE4 carriers even before cognitive decline.^40^ When BBB permeability or transport is impaired, the compensatory role of the perivascular ISF pathway might increase with the enlargement of perivascular spaces to improve fluid flow. As BBB transport is not related to tau removal, compensatory facilitation of perivascular ISF drainage accompanying EPVS may increase tau clearance, which results in lower tau deposition levels in the brain. By contrast, given that Aβ proteins are cleared via both BBB transport and the perivascular ISF pathway, the compensatory facilitation of the perivascular ISF pathway in response to BBB damage is not likely to significantly change the overall brain Aβ clearance and eventually Aβ deposition in the brain. We observed a significant negative association between EPVS and tau deposition only in BG‐EPVS, but not in CSO‐EPVS. A recent neuroimaging study provides a possible clue for the explanation of our results.^41^ The study showed that compromised BBB integrity, measured using dynamic contrast‐enhanced‐MRI, was associated with the severity of BG‐EPVS but not with that of CSO‐EPVS. These findings suggest that BBB dysfunction may be involved in the pathogenesis of BG‐EPVS, but not CSO‐EPVS.

Only a couple of studies have previously investigated the association between EPVS and cerebral tau pathology. In contrast to our results, Gertje et al.^13^ reported no significant association between BG‐EPVS and CSF tau levels. Besides the discrepancy in the measurement method for tau pathology, that is, measurement of tau level in CSF vs. measurement of tau deposition by PET, the differences in the study participants may explain the discordant results. The previous study by Gertje et al^13^ included only CN (67.5%) and MCI (22.5%) individuals, but not patients with dementia, to analyze the relationship between EPVS and CSF tau levels, while we included individuals with a diverse cognitive spectrum (CN [41.7%], MCI [27.6%], and Alzheimer's disease dementia [30.7%]). Our additional exploratory analysis for the moderation of the cognitive status showed that the relationship between BG‐EPVS and lower tau deposition was prominent in the CI but not in the CN. Given this, the null finding from the previous study might be related to the fact that more than two‐thirds of the participants were CN individuals, and less than one‐third were MCI individuals with no dementia. Similarly, another study on only CN individuals also reported no significant association between BG‐EPVS and tau deposition on PET.^14^ The association between more BG‐EPVS and lower tau pathology specifically observed only in the CI status may be explained by the fact that BBB integrity, related to BG‐EPVS,^41^ is more impaired in Alzheimer's disease dementia or MCI,^38^, ^42^, ^43^, ^44^ compared with the cognitively intact state.

Similarly, a significant association between BG‐EPVS and tau was observed only in the Aβ or APOE4‐positive subgroup. Aβ deposition is the upstream pathology of Alzheimer's disease‐specific neurodegeneration and cognitive impairment.^45^ APOE4, a major genetic risk of Alzheimer's disease dementia^46^ and Aβ deposition,^28^, ^47^ is known to accelerate BBB breakdown and degeneration of pericytes that maintain BBB integrity.^48^ Taken together, the significant association between BG‐EPVS and tau observed only in Aβ‐positive or APOE4‐positive cases can possibly be explained by BBB breakdown and related compensatory enlargement of the BG perivascular space as described above.

The CI individuals had more prevalent BG‐EPVS than the CN individuals in the present study, in line with previous reports that showed increased overall EPVS^7^ or BG‐EPVS^8^ in Alzheimer's disease dementia and MCI.^7^ Given this, together with the association between more BG‐EPVS and lower tau deposition, especially in CI or Aβ‐positive individuals, our results suggest that, while overall BG‐EPVS burden increases with cognitive decline or progression of Alzheimer's disease, elevated BG‐EPVS themselves may reduce tau pathology by facilitating clearance of tau proteins under CI conditions.

Our finding on the relationship between high BG‐EPVS and reduced cerebral tau burden is a novel observation. Nevertheless, some limitations of the current study need to be mentioned. First, as this was a cross‐sectional study, it was not possible to infer a causal relationship between EVPS and in vivo AD pathology. Further investigations are needed to clarify the mechanism underlying the association between increased BG‐EPVS and low tau deposition. Although a compensatory increase in the perivascular ISF pathway for tau elimination in response to compromised BBB permeability might be a possible explanation for the mechanism, we did not evaluate BBB permeability. Large longitudinal studies including dynamic contrast‐enhanced MRI for the measurement of BBB permeability can be helpful in overcoming the limitations of the present study.

In conclusion, our findings suggest that brain tau pathology appears to be reduced with increased BG‐EPVS, especially in individuals with cognitive impairment, pathological amyloid burden, or genetic Alzheimer's disease risk. Further investigations on the mechanism underlying the relationship between high BG‐EPVS and lower tau pathology is needed.

## AUTHOR CONTRIBUTIONS

KMK and DYL contributed to the conception and design of the study. KMK and DYL contributed to drafting the text and preparing figures. All authors contributed to the acquisition and analysis of data. MSB, DY, KHL, MJK, HA, GJ, JYL, YKK, YSL, and CHS contributed the acquisition, analysis and interpretation of data.

## FUNDING INFORMATION

This study was supported by grants from the Ministry of Science, ICT, and Future Planning, the Republic of Korea (NRF‐2014M3C7A1046042), the Ministry of Health & Welfare, Republic of Korea (HI18C0630 & HI19C0149), Seoul National University Hospital, the Republic of Korea (No. 3020200030), the National Institute of Aging, the United States of America (U01AG072177), the SNUH Research Fund (No. 0420210750), the Korea Medical Device Development Fund grant funded by the Korea government (the Ministry of Science and ICT, the Ministry of Trade, Industry and Energy, the Ministry of Health & Welfare, the Ministry of Food and Drug Safety) (No. 9991006735, KMDF_PR_20200901_0062), the National Research Foundation of Korea (NRF) grant funded by the Korea government (MSIT) (No. 2021R1C1C1006407), and the Brain Research Program through the NRF of Korea funded by the Ministry of Science, ICT & Future Planning (No. 2018M3C7A1056888). The funding source had no role in the study design, data collection, data analysis, data interpretation, writing of the manuscript, or decision to submit it for publication.

## CONFLICT OF INTEREST

None.

## Supporting information


Figure S1.

## Data Availability

The datasets generated and analyzed during the present study are not publicly available, owing to ethics considerations and privacy restriction. Data may be available from the corresponding author once approval from the Institutional Review Board of the Seoul National University Hospital, South Korea has been sought.
