# Supplementary material for: Enlarged perivascular spaces are associated with decreased brain tau deposition
Source: CNS Neurosci Ther. 2022 Dec 5;29(2):577–86. doi: 10.1111/cns.14040 (PMC9873511; doi:10.1111/cns.14040)
Supplement: Supplementary file 1 — Figure S1. [file CNS-29-577-s001.docx]

**Figure S1.**

Prevalence of high degree EPVS in CN and CI individuals.

(A) BG-EPVS. (B) CSO-EPVS.

* *p* < 0.05.

CN, cognitively normal; CI, cognitively impaired; BG-EPVS, enlarged perivascular spaces in the basal ganglia; CSO-EPVS, enlarged perivascular spaces in the centrum semiovale.

**
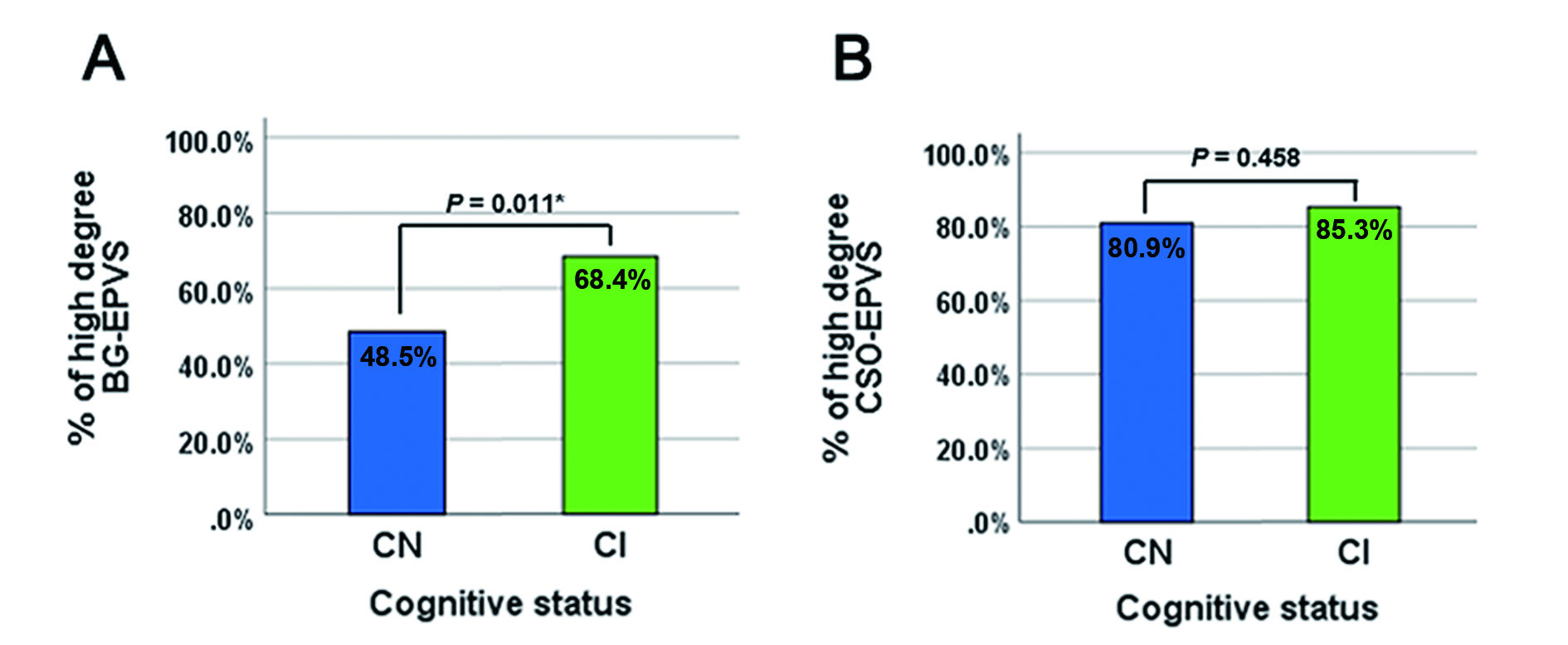
**

Figure S2.

Correlation between BG-EPVS and cerebral tau deposition in CI.


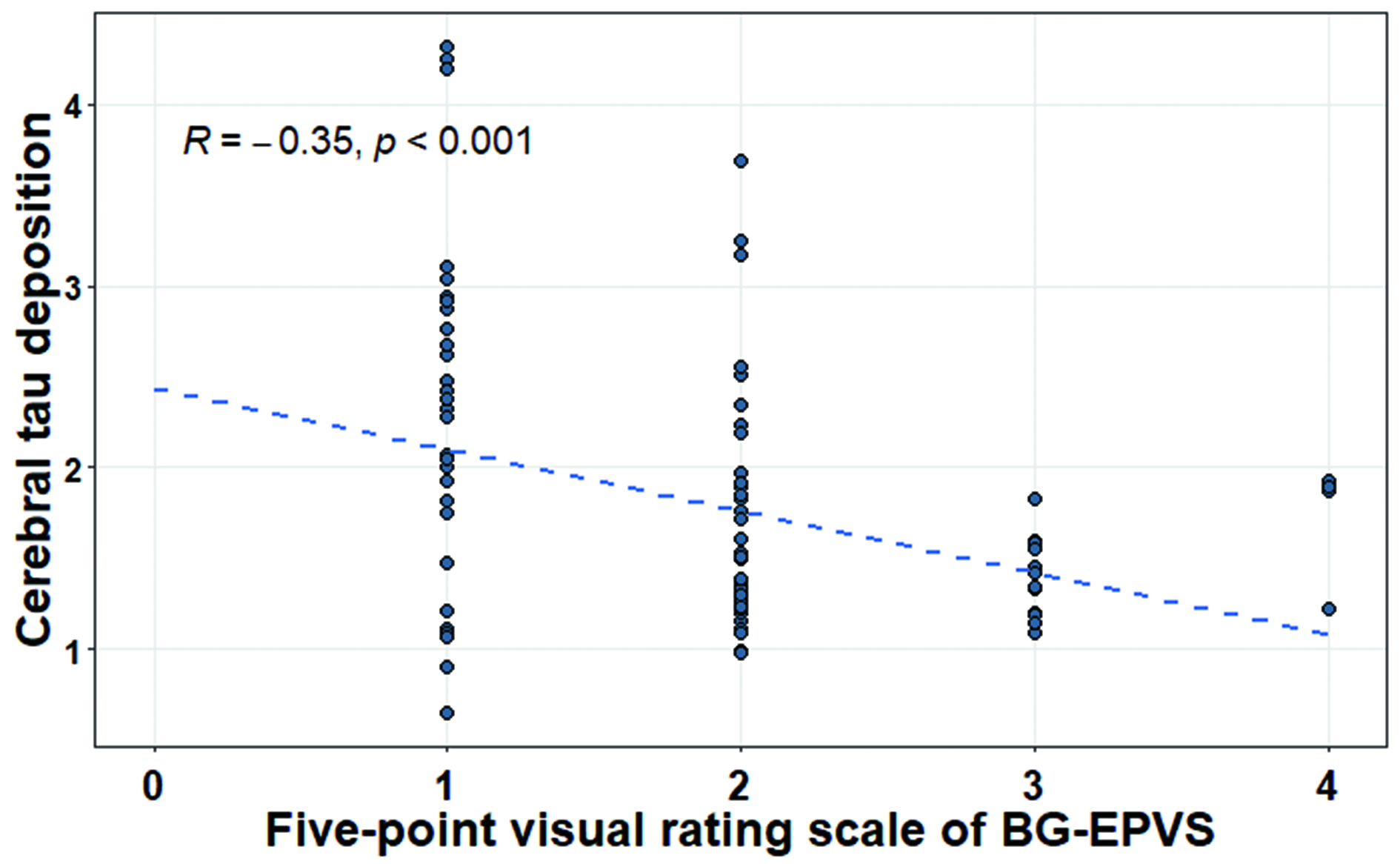


BG-EPVS, enlarged perivascular spaces in the basal ganglia; CI, cognitively impaired
